# Possible New Strategies for the Treatment of Congenital Hyperinsulinism

**DOI:** 10.3389/fendo.2020.545638

**Published:** 2020-10-27

**Authors:** Jelena Sikimic, Theresa Hoffmeister, Anne Gresch, Julia Kaiser, Winfried Barthlen, Carmen Wolke, Ilse Wieland, Uwe Lendeckel, Peter Krippeit-Drews, Martina Düfer, Gisela Drews

**Affiliations:** ^1^Department of Pharmacology, Institute of Pharmacy, University of Tübingen, Tübingen, Germany; ^2^Department of Pharmacology, Institute of Pharmaceutical and Medicinal Chemistry, University of Münster, Münster, Germany; ^3^Department of Pediatric Surgery, University Medicine Greifswald, Greifswald, Germany; ^4^Institute of Medical Biochemistry and Molecular Biology, University Medicine Greifswald, Greifswald, Germany; ^5^Institute of Human Genetics, University Hospital Magdeburg, Magdeburg, Germany

**Keywords:** congenital hyperinsulinism, K_ATP_ channels, diazoxide, NN414, L-type Ca^2+^ channels, K_Ca_3.1 channels

## Abstract

**Objective:**

Congenital hyperinsulinism (CHI) is a rare disease characterized by persistent hypoglycemia as a result of inappropriate insulin secretion, which can lead to irreversible neurological defects in infants. Poor efficacy and strong adverse effects of the current medications impede successful treatment. The aim of the study was to investigate new approaches to silence β-cells and thus attenuate insulin secretion.

**Research Design and Methods:**

In the scope of our research, we tested substances more selective and more potent than the gold standard diazoxide that also interact with neuroendocrine ATP-sensitive K^+^ (K_ATP_) channels. Additionally, K_ATP_ channel-independent targets as Ca^2+^-activated K^+^ channels of intermediate conductance (K_Ca_3.1) and L-type Ca^2+^ channels were investigated. Experiments were performed using human islet cell clusters isolated from tissue of CHI patients (histologically classified as pathological) and islet cell clusters obtained from C57BL/6N (WT) or SUR1 knockout (SUR1^-/-^) mice. The cytosolic Ca^2+^ concentration ([Ca^2+^]_c_) was used as a parameter for the pathway regulated by electrical activity and was determined by fura-2 fluorescence. The mitochondrial membrane potential (ΔΨ) was determined by rhodamine 123 fluorescence and single channel currents were measured by the patch-clamp technique.

**Results:**

The selective K_ATP_ channel opener NN414 (5 µM) diminished [Ca^2+^]_c_ in isolated human CHI islet cell clusters and WT mouse islet cell clusters stimulated with 10 mM glucose. In islet cell clusters lacking functional K_ATP_ channels (SUR1^-/-^) the drug was without effect. VU0071063 (30 µM), another K_ATP_ channel opener considered to be selective, lowered [Ca^2+^]_c_ in human CHI islet cell clusters. The compound was also effective in islet cell clusters from SUR1^-/-^ mice, showing that [Ca^2+^]_c_ is influenced by additional effects besides K_ATP_ channels. Contrasting to NN414, the drug depolarized ΔΨ in murine islet cell clusters pointing to severe interference with mitochondrial metabolism. An opener of K_Ca_3.1 channels, DCEBIO (100 µM), significantly decreased [Ca^2+^]_c_ in SUR1^-/-^ and human CHI islet cell clusters. To target L-type Ca^2+^ channels we tested two already approved drugs, dextromethorphan (DXM) and simvastatin. DXM (100 µM) efficiently diminished [Ca^2+^]_c_ in stimulated human CHI islet cell clusters as well as in stimulated SUR1^-/-^ islet cell clusters. Similar effects on [Ca^2+^]_c_ were observed in experiments with simvastatin (7.2 µM).

**Conclusions:**

NN414 seems to provide a good alternative to the currently used K_ATP_ channel opener diazoxide. Targeting K_Ca_3.1 channels by channel openers or L-type Ca^2+^ channels by DXM or simvastatin might be valuable approaches for treatment of CHI caused by mutations of K_ATP_ channels not sensitive to K_ATP_ channel openers.

## Introduction

Congenital hyperinsulinism (CHI) is a rare heterogeneous genetic disorder, but the most frequent cause of severe, persistent hypoglycemia in neonates, infants and children. The main reasons for developing CHI are defects in important genes regulating pancreatic β-cell function. To date, mutations in 14 essential genes controlling insulin secretion have been reported including *ABCC8* and *KCNJ11*. *ABCC8* and *KCNJ11* genes encode the K_ATP_ channel subunits SUR1 and Kir6.2, respectively, and mutations in these genes represent the most prevalent cause of CHI. Defects in these genes are responsible for the failure of β-cells to respond to normal regulatory mechanisms, leading to inappropriate and excessive insulin release despite low blood glucose concentrations resulting in frequent episodes of hypoglycemia (1, 2). There are some excellent reviews giving detailed information about molecular mechanisms underlying the pathophysiology of CHI (1–5).

Based on histopathological observations, three distinct forms of CHI are described: focal, diffuse and atypical. In focal CHI affected β-cells are localized only in small specific parts of the pancreas. Conversely, in diffuse CHI all pancreatic β-cells seem to be affected (6). If the histology of the tissue does not fit in one of the forms, it is regarded as an atypical form of CHI. It is characterized by a mosaic-like assembly of hyper-functional islets spread over the pancreas (7).

Persistent hypoglycemia is responsible for seizures and finally for severe brain damage (8). Thus, it is necessary to diagnose CHI rapidly and to start as early as possible with a suitable treatment. Treatment options include medical therapy and surgical intervention (9). First-line drug for treating CHI is the K_ATP_ channel agonist diazoxide (10). However, numerous side effects of diazoxide limit its use. Some of the most common undesired effects are Na^+^ and fluid retention, hypertrichosis and loss of appetite. Life threatening side effects also occur including cardiac failure, pulmonary hypertension, hyperuricemia, bone marrow suppression, and anemia (11–16). Additionally, diazoxide is only effective when K_ATP_ channels are functional (10). Alternatives to the therapy with diazoxide and novel medications include glucagon, somatostatin analogues, nifedipine, GLP1-receptor antagonists [exendin-(9–39)], and sirolimus [(17–22), reviewed in (3)]. Many of these drugs act by lowering the Ca^2+^ influx into β-cells (23–25). The aforementioned drugs also have numerous undesirable effects, which may be a reason for reconsidering their therapeutic usefulness: gastrointestinal symptoms, formation of gall stones, suppression of pituitary hormones, necrotizing enterocolitis, hypotension, immune suppression, thrombocytosis, impaired immune response, and many more (26–31). Recently, a new full human monoclonal antibody to the insulin receptor XMetD (also known as XOMA 358 or RZ358) has been proposed as a novel therapeutic strategy (32–35). First results in a Phase 2a clinical trial exhibited an improved glycemic control in patients with persistent hypoglycemia (36).

In patients that cannot be treated sufficiently with drugs, surgical treatment is indicated. While partial pancreatectomy is beneficial for patients with focal CHI (37, 38), in case of diffuse and drug-unresponsive CHI, near-total pancreatectomy is usually required (39, 40). Due to different post-operative complications like recurrent hypoglycemia, pancreatic exocrine insufficiency and diabetes, patients with diffuse CHI are far from being cured after surgery (41, 42). In order to reduce the development of diabetes postsurgically, a 70 to 90% resection of pancreas have been considered; however, the outcome is still unpredictable (39, 43).

Taken together, it is of great importance to explore new pharmacological options for CHI therapy in order to maintain euglycemia and reduce severe side effects from current medical and surgical treatment. Aim of this study was to find new strategies, which are able to silence β-cells by inhibiting extensive Ca^2+^ influx into the cell. For this purpose, new and approved drugs interacting with K_ATP_ channels and with K_ATP_ channel-independent targets have been tested on islet cell clusters obtained from biopsies of CHI patients and islet cell clusters from WT and SUR1^-/-^ mice.

## Materials and Methods

### Cell and Islet Preparation

Human islets of Langerhans were obtained from different biopsies of children undergoing pancreatic surgery. Ethics approval for the study involving human participants was approved by the ethic commission of the Universitätsmedizin Greifswald (BB 050/13). Written informed consent was provided by the legal guardians of the children for the study. The islets were taken from biopsies of eight CHI patients. Genetic studies showed that seven patients had mutations in the *ABCC8* gene encoding the SUR1 subunit of K_ATP_ channels. In one biopsy no mutation was found for eight genes tested (**Table 1**: 2). According to postsurgical evaluation of the biopsies by the Department of Pathology at the University Hospital Greifswald, the tissue was identified as pathological and assigned to the CHI type (mosaic, diffuse or focal). Islets of these pathological samples were isolated by injecting collagenase (2–4 mg/ml) into the biopsy material and by handpicking islets after digestion at 37°C. Afterward, islets were cultured in a CMRL 1066 medium with 5.5 mM glucose supplemented with 10% fetal calf serum, 100 U/ml of penicillin, 100 mg/ml of streptomycin, 10 mM HEPES, and 2 mM l-glutamine. Next day, the samples were shipped to Tübingen and/or Münster for further analysis.

**Table 1:** Genetic characteristics of patients.

Pat. No.	Age at surgery (months)	Gene	Nucleotide position	Protein effect	Mutation type	Gene	Zygosity	Inheritance	Diazoxide response (literature)	Diazoxide response (individual clinical data)	Form	Reference
**1**	1-6	*ABCC8*	c.4435G > A	p.(Gly1479Arg)	missense	exon 37	heterozygote	dom/paternal	(yes)	partial	mosaic	*Nichols et al. (*44*); Pinney et al. (*45*); Sandal et al. (*46*); Kapoor et al. (*47*); Snider et al. (*48*)*
**2**	12-24	None*	N/A	N/A	N/A	N/A	N/A	N/A	N/A	partial	diffuse	N/A
**3**	6-12	*ABCC8*	c.3992-9G>A	p.0	splicing	intron 32	heterozygote	rec/paternal	(no)	partial	focal	*Nestorowicz et al. (*49*); Nestorowicz et al. (*50*); Arya et al. (*51*)*
**4**	6-12	*ABCC8*	c.3970G>T	p.(Glu1324*)	nonsense	exon 32	heterozygote	rec/paternal	N/A	partial	focal	*De Franco et al. (*52*)*
**5**	6-12	*ABCC8*	c.2509C>T	p.(Arg837*)	nonsense	exon 21	hetreozygote	rec/paternal	no	no	focal	*Craig et al. (*53*); Park et al. (*54*); Kapoor et al. (*55*); Craigie et al. (*53*); Snider et al. (*48*)*
**6**	24-36	*ABCC8*	c.1176G>C	p.(Gln392His) p.?	missense / splicing	exon 7	homozygote	rec/bi-parental	partial	N/A	diffuse	*Ince et al. (*56*); Corda et al. (*57*)*
**7**	12-24	*ABCC8*	c.1183 A>T c.4146T>G	p.(Ile395Phe) p.(Ser1382Arg)	missense	exon 8 exon 34	compound heterozygote	maternal / de novo	(yes)	no	diffuse	*De Franco et al. (*52); ClinVar** ID265990
**8**	1-6	*ABCC8*	N/A	N/A	N/A	N/A	N/A	suspected paternal	N/A	no	focal	N/A

*No mutation was found in 8 CHI genes; **National Center for Biotechnology Information. ClinVar; [VCV000265990.1], https://www.ncbi.nlm.nih.gov/clinvar/variation/VCV000265990.1 (accessed July 12, 2020).

The diazoxide response column indicates the response to diazoxide according to literature and brackets are used to note on exceptions.

N/A, not available; dom, dominant; rec, recessive.

Mouse islets of Langerhans were isolated from adult C57BL/6N (WT) mice or SUR1 knockout (SUR1^-/-^) mice on a C57BL/6N background. The mice were bred in the animal facility of the Department of Pharmacology at the University of Tübingen. The principles of laboratory animal care (NIH publication no. 85-23, revised 1985) and German laws were followed. The animal study was reviewed and approved by the Regierungspräsidium Tübingen (§ 4 Abs. 3 TierSchG). Islets were isolated and cultured as previously described (58).

For experiments, human or mouse islet cell clusters of similar size were used, obtained by dispersing islets by trypsin treatment. Human and mouse islet cell clusters were kept in cell culture up to 3 days.

### Solutions and Chemicals

Measurements of [Ca^2+^]_c_ were performed with a bath solution, which contained (in mM): 140 NaCl, 5 KCl, 1.2 MgCl_2_, 2.5 CaCl_2,_ 10 HEPES and glucose as indicated, pH 7.4 adjusted with NaOH. The same bath solution was used for the determination of the mitochondrial membrane potential (ΔΨ). The pipette solution for single channel recording contained (in mM): 130 KCl, 1.2 MgCl_2_, 2 CaCl_2_, 10 EGTA, and 10 HEPES; pH was adjusted to 7.4 with KOH. The bath solution contained (in mM): 130 KCl, 2 CaCl_2_, 10 EGTA, 1 Na_2_ATP, 1.7 MgCl_2_, and 20 HEPES with pH adjusted to 7.2 with KOH.

NN414, diazoxide and simvastatin were obtained from Sigma-Aldrich (Schnelldorf, Germany). DCEBIO was either purchased from Tocris Bioscience (Bristol, United Kingdom) or Santa Cruz (Heidelberg, Germany), fura-2-AM from Biotrend (Köln, Germany), and dextromethorphan (DXM) from Alfa Aesar (Kandel, Germany). Rhodamine 123 (Rh123), RPMI 1640 medium, CMRL 1066 medium, Dulbecco's modified Eagle's medium, fetal calf serum (FCS), penicillin/streptomycin, glutamine, and trypsin were from Invitrogen (Karlsruhe, Germany). Collagenase used for human biopsy material was obtained from Roche Diagnostics GmbH (Mannheim, Germany). All other chemicals were obtained from Sigma-Aldrich or Carl Roth (Karlsruhe, Germany) in the purest form available.

### Measurements of [Ca^2+^]_c_

Details are described in (58). In brief, islet cell clusters were loaded with 5 µM fura-2-AM for 30-35 min at 37°C. The cells were perifused with bath solution with the indicated test substances. Fluorescence was excited at 340 and 380 nm, emission was filtered (LP515) and measured by a digital camera. Cytosolic Ca^2+^ concentration was measured as the ratio of the fluorescence intensities (F340/F380) of the emitted light excited with 340 nm and 380 nm. A ratio, i.e., one data point, was measured every 3 s. Ca^2+^ in glucose-activated beta cells oscillates between a basal and a maximal concentration. Decisive for insulin secretion is the mean Ca^2+^ concentration. Therefore, the data points were averaged 5–8 min before the end of a maneuver, to compare [Ca^2+^]_c_ under different experimental conditions.

### Measurements of the Mitochondrial Membrane Potential (ΔΨ)

ΔΨ was measured by Rh123 fluorescence at 480 nm excitation wavelength as described in (59). One data point was measured every 3 s. The effects were evaluated by averaging the values of the last 60 s of each interval before solution change. At the end of each experiment FCCP (0.5 µM) was added to evaluate maximal mitochondrial depolarization. Rh123 fluorescence corresponds to the proton gradient across the inner mitochondrial membrane and thus to ATP production. A decrease in fluorescence indicates a hyperpolarization and an increase in ATP production and *vice versa*.

### Patch-clamp recordings

Hamster cDNA encoding for the SUR1_E1507K_ protein together with WT human cDNA for Kir6.2 was expressed in a stably transfected HEK-293 cell line (60). Cells were cultured in Dulbecco’s modified Eagle’s medium supplemented with 10% FCS, glutamine, 100 U/ml of penicillin, and 100 mg/ml of streptomycin. Expression was induced by addition of doxycycline (300 µM) and cells were used for characterization of channel activity from 24 to 72 h.

Patch-clamp recordings were done in the inside-out configuration. K_ATP_ currents were measured at a membrane potential of -50 mV (pipette voltage, +50 mV); inward currents are shown as downward deflections. Patch pipettes had a resistance of 6–8 MΩ. Currents were recorded with an EPC-9 patch-clamp amplifier using Patchmaster software (HEKA, Lambrecht, Germany). Analyses to estimate mean current were done offline in IgorPro 7 (Wavemetrics, Inc., Lake Oswego, OR). With diazoxide or NN414 so many channels open simultaneously that a single channel evaluation of open probability (Po) was not possible. We therefore evaluated the mean current for 20 s before the end of a maneuver.

### Statistics

Each series of experiments with islet cell clusters from mice was performed with at least three independent mouse preparations. The number of preparations for recordings with human islet cell clusters varied and is indicated for every series. Box plots were generated using Graphpad Prism 8. Boxes correspond to the interquartile range, the line within the box to the median, and the cross to the mean. Whiskers correspond to the maximum and minimum values. Statistical significance of differences was assessed by Student’s *t* test. Multiple comparisons were made by ANOVA followed by Student-Newman-Keuls test. P values ≤ 0.05 were considered significant.

## Results

### Effects of Nifedipine on the Cytosolic Ca^2+^ Concentration in Human Islet Cell Clusters

**Figure 1:**
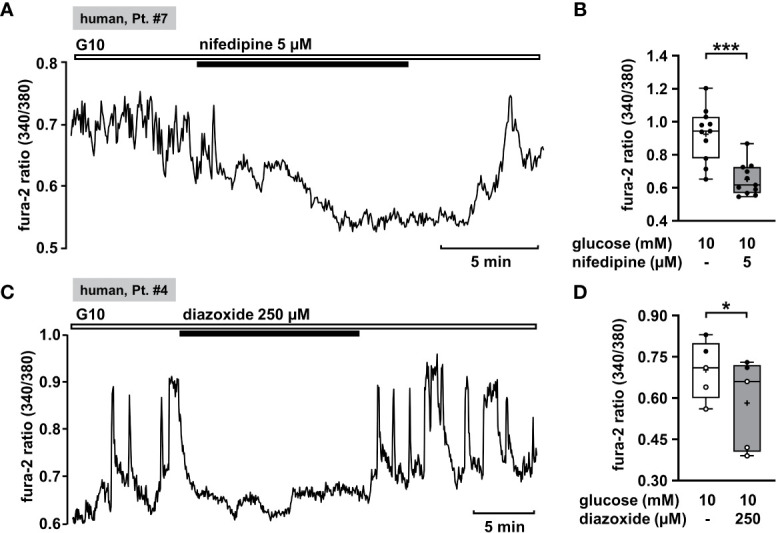
Nifedipine and diazoxide reduce [Ca^2+^]_c_ in human CHI islet cell clusters. **(A)** Representative recording showing inhibition of glucose-induced oscillations of [Ca^2+^]_c_ by nifedipine (5 µM) in the presence of 10 mM glucose in a human islet cell cluster isolated from pancreatic tissue of a patient with diffuse CHI (**Table 1**: 7, depicted as “human, Pt. #7” in the figure). **(B)** Summary of all experiments recorded in the presence of 10 mM glucose comprising 11 islet cell clusters isolated from pancreatic tissue of one CHI patient (**Table 1**: 7). **(C)** Representative recording showing the influence of diazoxide (250 µM) on glucose-induced oscillations of [Ca^2+^]_c_ in the presence of 10 mM glucose in a human islet cell cluster isolated from pancreatic tissue of a patient with focal CHI (**Table 1**: 4). **(D)** Summary of all respective experiments from two patients, one with focal and one with mosaic form of CHI (**Table 1**: 1, black circles; 4, white circles) (n = 5). *p ≤ 0.05 and ***p ≤ 0.001.

Oscillations of the cytosolic Ca^2+^ concentration ([Ca^2+^]_c_) are driven by fluctuations of the membrane potential and [Ca^2+^]_c_ is the trigger signal for insulin secretion. Consequently, [Ca^2+^]_c_ is a very robust surrogate parameter for insulin secretion. It can be determined easily and online with few cell material, which is an enormous advantage when working with human tissue. Glucose-stimulated insulin secretion in human pancreatic β-cells is completely suppressed by pharmacologic blockage of L-type Ca^2+^ channels (61). This mechanism should also be functional in CHI islet cells. Thus, as control we tested the effect of the L-type Ca^2+^ channel blocker nifedipine on [Ca^2+^]_c_ in human islet cell clusters isolated from tissue of a patient with diffuse CHI (**Table 1**: 7). **Figure 1A** presents a recording with fast oscillations of [Ca^2+^]_c_ on top of a plateau in the presence of a stimulating glucose concentration of 10 mM. The addition of nifedipine at a concentration of 5 µM diminished [Ca^2+^]_c_ significantly (**Figure 1B**).

### K_ATP_ Channel Openers

#### Effects of Diazoxide on [Ca^2+^]_c_ of Human Islet Cell Clusters

K_ATP_ channels (SUR1/Kir6.2) of pancreatic β-cells play a crucial role as they couple cellular metabolism to electrical activity. In electrically inactive β-cells, K_ATP_ channels are open. CHI is characterized by permanently active β-cells and thus opening of these channels is one strategy to treat it. Diazoxide is an opener of K_ATP_ channels that is already established in CHI therapy. We tested the effect of diazoxide on [Ca^2+^]_c_ as a control. In human islet cell clusters isolated from two patients, one with focal and one with mosaic form of CHI (**Table 1**: 1 and 4), 250 µM diazoxide clearly decreased the mean fluorescence ratio (**Figures 1C, D**). These results show that the channels of these patients are in principle functional and can be influenced by the K_ATP_ channel opener, although mutations in the *ABCC8* gene were reported to be the cause of CHI. Obviously, the complex regulation of the channels is disturbed, e.g., the sensitivity to MgATP (1).

#### Effects of NN414 on [Ca^2+^]_c_ of Human and Mouse Islet Cell Clusters and Mitochondrial Membrane Potential of Mouse Islet Cell Clusters

**Figure 2:**
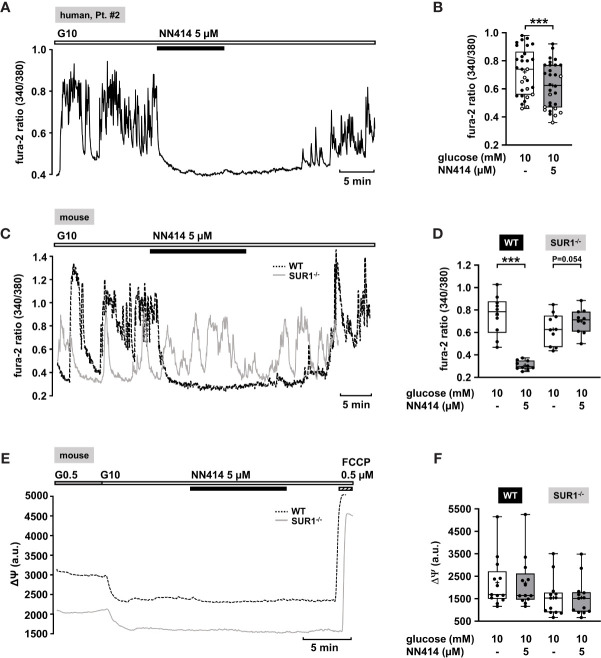
Effects of NN414 on [Ca^2+^]_c_ and mitochondrial membrane potential ΔΨ. **(A)** Representative recording showing the reduction of glucose-induced oscillations of [Ca^2+^]_c_ by NN414 (5 µM) in the presence of 10 mM glucose in a human islet cell cluster isolated from pancreatic tissue of a patient with diffuse CHI (**Table 1**: 2). **(B)** Summary of all respective experiments from three patients, one with diffuse, one with focal, and one with mosaic form (**Table 1**: 1, black circles; 2, white circles; and 4, gray circles) (n = 30). **(C)** Representative recordings showing the effect of NN414 (5 µM) on glucose-induced oscillations of [Ca^2+^]_c_ in islet cell clusters from WT (dashed curve) and SUR1^-/-^ (gray curve) mice. NN414 significantly reduced [Ca^2+^]_c_ in islet cell clusters from WT mice, but not in islet cell clusters from SUR1^-/-^ mice. **(D)** Summary of all respective experiments (n = 10 for each genotype, three different mouse preparations for each series). **(E)** Typical recordings showing measurement of ΔΨ in islet cell clusters obtained from WT (dashed curve) and SUR1^-/-^ (gray curve) mice. The switch from 0.5 to 10 mM glucose hyperpolarizes ΔΨ. The addition of NN414 has no influence on ΔΨ in WT and SUR^-/-^ islet cell clusters, respectively. **(F)** Summary of all experiments made under these conditions (n = 13, three different mouse preparations for each series). ***p ≤ 0.001.

The diazoxide analogue NN414 is suggested to be a selective agonist of pancreatic β-cell K_ATP_ channels, and is 100-fold more potent than diazoxide (62). Therefore, it has been proposed as useful drug for the treatment of diseases with excessive insulin secretion (62). NN414, in a concentration of 5 µM, completely abolished oscillations of [Ca^2+^]_c_ and reduced [Ca^2+^]_c_ to basal levels (**Figure 2A**). Application of 5 µM NN414 to human CHI islet cell clusters taken from three different forms (focal, diffuse, and atypical mosaic, **Table 1**: 1, 2, and 4) significantly lowered the mean fluorescence ratio (**Figure 2B**).

To test whether NN414 specifically interferes with K_ATP_ channels, we studied the effects of this compound on [Ca^2+^]_c_ with islet cell clusters of WT mice and mice lacking functional K_ATP_ channels (SUR1^-/-^ mice) (63). As expected, 5 µM NN414 abolished [Ca^2+^]_c_ oscillations in islet cell clusters of WT mice (**Figure 2C**, black trace) and reduced the mean fluorescence ratio (**Figure 2D**, left part). By contrast, NN414 hardly affected [Ca^2+^]_c_ oscillations and did not decrease the mean fluorescence ratio in islet cell clusters obtained from SUR1^-/-^ mice (**Figures 2C**, gray trace, **2D**, right part).

Some K_ATP_ channel openers affect mitochondrial function in addition to their direct influence on K_ATP_ channels (64). To address this point, comparative experiments with islet cell clusters of the two mouse genotypes were performed evaluating a possible effect of NN414 on the mitochondrial membrane potential. **Figure 2E** shows typical recordings of ΔΨ for a WT and a SUR1^-/-^ islet cell cluster. The switch from 0.5 to 10 mM glucose is accompanied by a strong decrease in Rh123 fluorescence reflecting hyperpolarization of ΔΨ and thus ATP production upon the increase of glucose concentration (65, 66). This maneuver was performed in each cell cluster to test for glucose responsiveness. At the end of each experiment, the uncoupler FCCP was applied to evaluate maximal mitochondrial depolarization. Neither in WT nor in islet cell clusters from SUR1^-/-^ mice NN414 (5 µM) exerted any effect on ΔΨ (**Figure 2F**).

#### Diazoxide and NN414 Open K_ATP_ Channels Carrying a CHI Mutation

**Figure 3:**
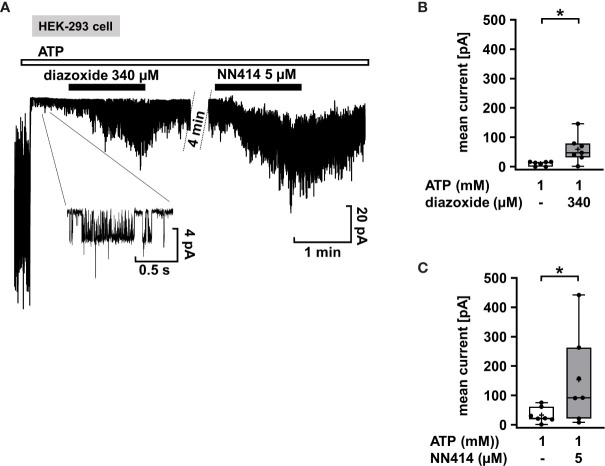
Diazoxide and NN414 open mutated K_ATP_ channels. **(A)** Representative trace showing the activation of SUR1_E1507K_/WT Kir 6.2 channels expressed in HEK-293 cells by the channel agonists diazoxide and NN414. This mutation is associated with CHI. At the beginning of the experiment the patch was pulled in nucleotide-free medium, which activates numerous channels as inhibitory nucleotides leave the pore. Addition of ATP rapidly inhibits almost all channel activity. Concurrent application of diazoxide (340 µM) or NN414 (5 µM) enhances channel activity. **(B**, **C)** Summary of all experiments with diazoxide (n = 7) and NN414 (n = 7), respectively. *p ≤ 0.05. The inset shows single channel openings at extended scales. The channel has an amplitude of about 4 pA, giving, at 50 mV driving force, a conductance of 80 pS, which is typical for K_ATP_ channels under these conditions. Four min under control conditions of the continuous recording were taken out for the clarity of the figure.

Mutations in the K_ATP_ channel subunits are the most common cause of CHI. However, they do not necessarily lead to diazoxide unresponsiveness. Response to diazoxide is even observed in patients in whom non-response would be predicted (67). Moreover, focal CHI is clinically heterogeneous and responsiveness or resistance to diazoxide was reported for patients with the same mutation in K_ATP_ channels (68). It is unclear whether these clinical effects are due to interference of diazoxide with the mutated channels or off-target effects. As an example illustrating the efficacy of K_ATP_ channel openers on mutant K_ATP_ channels, we used SUR1_E1507K_/WT Kir 6.2 channels since the SUR_E1507K_ mutation leads to CHI (69), but patients with this Glu to Lys mutation respond well to diazoxide (45). SUR1_E1507K_/WT Kir 6.2 channels were expressed in HEK-293 cells, and diazoxide was tested in comparison to NN414. **Figure 3A** shows that when inside/out patches from cells expressing SUR1_E1507K_/WT Kir 6.2 channels were pulled into nucleotide-free medium, numerous channels were activated as nucleotides inhibiting channel activity by antagonism on their WT pores were washed away (see start of the experiment before ATP application). Addition of 1 mM ATP rapidly inhibited channel activity as expected. Application of diazoxide (340 µM) or NN414 (5 µM) in the presence of ATP led to opening of SUR1_E1507K_/WT Kir 6.2 channels. (**Figures 3B, C**), showing that K_ATP_ channel agonists can directly affect mutated channels.

#### VU0071063 Silences Islet Cell Clusters in a K_ATP_ Channel-Dependent and -Independent Manner

**Figure 4:**
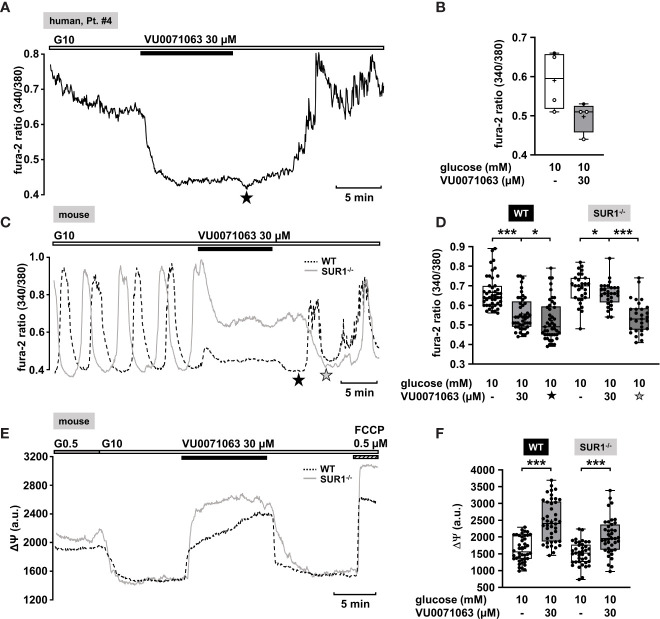
K_ATP_ channel-dependent and -independent effects of VU0071063. **(A)** Representative recording showing the reduction of [Ca^2+^]_c_ by VU0071063 (30 µM) in the presence of 10 mM glucose in a human islet cell cluster isolated from pancreatic tissue of a CHI patients with a focal lesion (**Table 1**: 4). The star depicts the nadir after wash-out of VU0071063. **(B)** Summary of four experiments obtained from two patients, one with focal and one with diffuse form of CHI. VU0071063 rapidly reduced [Ca^2+^]_c_ in all 4 experiments, but due to the low number of experiments, the effect is not significant. (**Table 1**: 2, black circles; 4, white circles). **(C)** Representative recordings showing the effect of VU0071063 (30 µM) on oscillations of [Ca^2+^]_c_ induced by 10 mM glucose in islet cell clusters from WT (dashed curve) and SUR1^-/-^ (gray curve) mice. VU0071063 significantly reduced [Ca^2+^]_c_ in islet cell clusters from SUR1^-/-^ mice, revealing K_ATP_ channel-independent effects of the compound. Note the drop in [Ca^2+^]_c_ after removal of VU0071063 (black star: WT, gray star: SUR1^-/-^). **(D)** Summary of all respective experiments; n = 45 and 29 for WT and SUR^-/-^ islet cell clusters. **(E)** Representative recordings showing the effect of VU0071063 (30 µM) on the mitochondrial membrane potential (ΔΨ) in islet cell clusters obtained from WT (dashed curve) and SUR1^-/-^ (gray curve) mice. **(F)** Summary of all respective experiments; n = 42 and 39 for WT and SUR^-/-^ islet cell clusters. Cell cluster were isolated from three WT and three SUR1^-/-^ mice. *p ≤ 0.05 and ***p ≤ 0.001.

Recently, Raphemot et al. discovered a novel xanthine derivative, VU0071063 that directly and selectively activates K_ATP_ channels (70). They found that VU0071063 is more potent and activates K_ATP_ channels with a faster kinetic than diazoxide. These findings encouraged us to test its effect on changes in [Ca^2+^]_c_ on human islet cell clusters from CHI patients. Administration of VU0071063 (30 µM) to islet cells clusters from pancreatic tissue with a focal or diffuse lesion (**Table 1**: 2 and 4) induced a prompt reduction of [Ca^2+^]_c_ in all four measurements. **Figure 4A** shows a typical example. The mean fluorescence ratio clearly changed (**Figure 4B**). Due to the limited pathological material, which explains the low number of experiments, we did not perform a statistical test with this data.

In the human islet cell clusters, a drop of [Ca^2+^]_c_ was noticed directly after withdrawal of VU0071063 (**Figure 4A**, asterisk). Presumably, this drop is due to ATP-dependent sequestration of Ca^2+^ into the ER (71). This suggests that VU0071063 affects additional targets besides K_ATP_ channels. To evaluate this assumption, [Ca^2+^]_c_ of islet cell clusters from WT mice and SUR1^-/-^ mice was measured. VU0071063 (30 µM) suppressed Ca^2+^ oscillations and lowered [Ca^2+^]_c_ in both genotypes (**Figures 4C–F**). Note that the effect was weaker in the cells of the knock-out mice. Like in human islet cell clusters, the drug further reduced [Ca^2+^]_c_ after its removal in both WT and SUR1^-/-^ mouse islet cell clusters (**Figure 4C**, asterisks). This points to alterations in mitochondrial metabolism, which can cause changes in K_ATP_ channel activity independent of any direct interaction with the channel proteins (72). As the mitochondrial membrane potential is for the most part directly linked to ATP production (65), we evaluated effects of VU0071063 on ΔΨ. Similar to the experiments described above, a rise in the glucose concentration caused a decrease of the fluorescence signal (**Figure 4E**). In islet cell clusters of WT mice and of SUR1^-/-^ mice 30 µM VU0071063 strongly and reversibly depolarized mitochondrial membrane potential (**Figures 4E, F**).

### K_ATP_ Channel-Independent Drugs

In the following part we present drugs and potential strategies, which could be effective in CHI patients non-responsive to K_ATP_ channel openers.

#### K_Ca_3.1 Channel Openers as a Potential Approach

In addition to K_ATP_ and voltage-gated K^+^ channels, pancreatic β-cells express K^+^ channels regulated by the cytosolic Ca^2+^ concentration (K_Ca_) (72). Depending on their single channel conductance, there are three groups whose existence has been detected in pancreatic β-cells (73–76). It has been demonstrated that the K_Ca_ channels of intermediate conductance (K_Ca_3.1, SK4) play an important role in the K^+^ current (K_slow_) that contributes to β-cell hyperpolarization at the end of a burst phase with electrical activity (66, 74, 77, 78). Previous results from Düfer et al. (74) demonstrated that activation of K_Ca_3.1 channels hyperpolarized the membrane potential of pancreatic β-cells from WT mice. Since about 50% of K_slow_ is K_ATP_ current (79), the sulfonylurea-insensitive K_Ca_ component could be even more significant in β-cells lacking functional K_ATP_ channels, which resembles the situation in CHI channelopathies.

**Figure 5:**
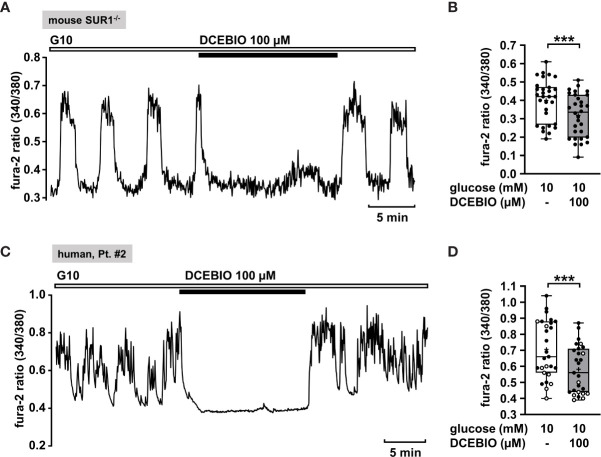
The K_Ca_3.1 channel opener DCEBIO reduces [Ca^2+^]_c_ in islet cell clusters isolated from SUR1^-/-^ mice and in human islet cell clusters. **(A)** Representative recording showing rapid inhibition of glucose-induced oscillations of [Ca^2+^]_c_ by DCEBIO (100 µM) in the presence of 10 mM glucose in islet cell clusters from SUR1^-/-^ mice. **(B)** Summary of all respective experiments; n = 30. Islet cell clusters were obtained from three different SUR1^-/-^ mice preparations. ***p ≤ 0.001. **(C)** Representative recording showing the reduction of glucose-induced oscillations of [Ca^2+^]_c_ by DCEBIO (100 µM) in the presence of 10 mM glucose in a human islet cell cluster isolated from pancreatic tissue affected by diffuse CHI (**Table 1**: 2). **(D)** Summary of all respective experiments from biopsies of two CHI patients, one with diffuse, one with mosaic form (**Table 1**: 1, black circles; 2, white circles) (n = 27). ***p ≤ 0.001.

To verify this assumption, we evaluated the effect of the K_Ca_3.1 opener DCEBIO on islet cell clusters isolated from SUR1^-/-^ mice. DCEBIO (100 µM) effectively abolished the glucose-induced oscillations of [Ca^2+^]_c_ (**Figure 5A**) and reduced the mean fluorescence ratio (**Figure 5B**). Next, we tested the effect of DCEBIO on human islet cell clusters from tissue of pancreatectomies. DCEBIO was tested on human islet cell clusters isolated from pancreatic tissues with mosaic and diffuse forms of CHI (**Table 1**: 1 and 2). The compound suppressed the oscillations of [Ca^2+^]_c_ (**Figure 5C**) and significantly decreased the mean fluorescence ratio (**Figure 5D**).

#### Effect of Dextromethorphan on [Ca^2+^]_c_ of Human Islet Cell Clusters

Dextromethorphan (DXM) is a known antagonist of NMDA receptors. Active NMDA receptors can activate other ion channels, like Ca^2+^-activated K^+^ channels or K_ATP_ channels and thus potentiate K^+^ outflow (80). A block of NMDA receptors leads to prolonged depolarization and increases insulin secretion (81). Lesser-known is its ability to directly inhibit L-type Ca^2+^ channels. Carpenter et al. found that DXM moderately inhibits L-type Ca^2+^ channels, thereby lowering [Ca^2+^]_c_. This effect was observed with permanently depolarized cells under stimulating glucose concentrations (82). Since permanent depolarization is a characteristic of CHI β-cells, DXM may offer a possibility to rescue, i.e., silence the overstimulated cells.

**Figure 6:**
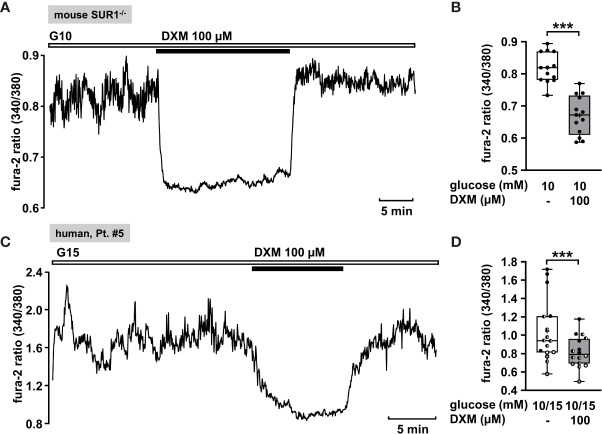
DXM lowers [Ca^2+^]_c_ in islet cell clusters lacking functional K_ATP_ channels. **(A)** Representative recording showing a rapid decrease of [Ca^2+^]_c_ by DXM (100 µM) in the presence of 10 mM glucose in an islet cell cluster from a SUR1^-/-^ mouse. **(B)** Summary of all respective experiments (n = 13) with different cell cluster from three SUR1^-/-^ mice. ***p ≤ 0.001. **(C)** Representative recording showing reduction of [Ca^2+^]_c_ by DXM (100 µM) in the presence of 15 mM glucose in a human islet cell cluster isolated from pancreatic tissue of a patient with focal CHI (**Table 1**: 5). **(D)** Summary of all respective experiments obtained from biopsies of two patients with focal and two patients with diffuse CHI (**Table 1**: 3, gray circles; 5, black circles; 6, white circles; and 7, hatched circles) (n = 16). ***p ≤ 0.001.

The measurement in **Figure 6A** shows a recording of [Ca^2+^]_c_ of a permanently depolarized islet cell cluster from a SUR1^-/-^ mouse in the presence of 10 mM glucose and application of 100 µM DXM. The drug significantly lowered the mean fluorescence ratio (**Figure 6B**). The mean fluorescence, application of DXM rapidly reduced [Ca^2+^]_c_ in an islet cell cluster from a patient with diffuse CHI (**Figure 6C**). The mean fluorescence ratio measured in islet cell clusters obtained from two patients with focal and two patients with diffuse CHI (**Table 1**: 3, 5, 6 and 7) was lowered (**Figure 6D**).

#### Statins as a Potential Strategy to Silence Human Islet Cell Clusters

Lipid-lowering statins are inhibitors of the enzyme HMG-CoA-reductase, which plays a significant role in cholesterol synthesis by converting HMG-CoA to mevalonate. For these drugs it has been reported that they increase the risk of type 2 diabetes (83). Different studies have been conducted in order to enlighten the mechanism how the statins impair insulin secretion. In the study using β-cells isolated from rats, Yada et al. (84) showed that simvastatin in a concentration of 3 µg/ml acutely blocked L-type Ca^2+^ channels, thus lowering insulin secretion. Furthermore, Yaluri et al. demonstrated that simvastatin diminished glucose-stimulated insulin secretion and [Ca^2+^]_c_ in MIN6 β-cells *via* multiple mechanisms (85).

**Figure 7:**
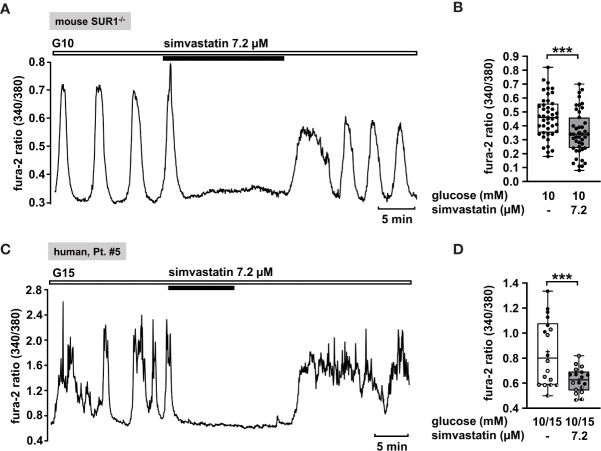
Simvastatin as a potential strategy to silence islet cell clusters affected by CHI. **(A)** Representative recording showing rapid inhibition of glucose-induced oscillations of [Ca^2+^]_c_ by simvastatin (7.2 µM) in the presence of 10 mM glucose in an islet cell cluster from SUR1^-/-^ mice. **(B)** Summary of all respective experiments (n = 43) with islet cell clusters obtained from three SUR1^-/-^ mice. ***p ≤ 0.001. **(C)** Representative recording showing the reduction of glucose-induced oscillations of [Ca^2+^]_c_ by simvastatin (7.2 µM) in the presence of 15 mM glucose in a human islet cell cluster isolated from pancreatic tissue of a patient affected by focal CHI (**Table 1**: 5). **(D)** Summary of all respective experiments from biopsies of two patients with focal and two patients with diffuse CHI (**Table 1**: 5, black circles; 6, white circles; 7, gray circles; and 8, hatched circles) (n = 18). ***p ≤ 0.001.

Hence, we considered simvastatin as a potential therapeutic strategy to treat CHI. In order to confirm that simvastatin shows its effect when functional K_ATP_ channels are lacking, we measured [Ca^2+^]_c_ in islet cell clusters from SUR1^-/-^ mice (**Figure 7A**). Simvastatin in a concentration of 7.2 µM [according to the concentration of 3 µg/ml that was used in the study of Yada et al. (84)] rapidly decreased the glucose-stimulated Ca^2+^ oscillations and diminished the mean fluorescence ratio (**Figure 7B**). Further, we tested simvastatin on human islet cell clusters. **Figure 7C** shows a representative measurement of [Ca^2+^]_c_ in an islet cell cluster isolated from pancreatic tissue of a patient with focal CHI. The mean fluorescence ratio markedly declined (**Figure 7D**). The biopsy material was obtained from two patients with focal and two patients with diffuse CHI (**Table 1**: 5 – 8).

## Discussion

### Possible KATP Channel-Dependent Strategies to Treat CHI

In the present study islet cell clusters isolated from biopsies of CHI patients were used to search for new strategies to treat the disease. Pancreatic islet cell clusters isolated from either focal, diffuse or atypical pancreatic tissue were used as material. We are aware that these are distinct diseases. Due to the limited material we did not separate our results according to the different CHI forms. Noteworthy, the aim of the study was not to suggest novel drugs for CHI treatment but to optimize existing approaches, to search for novel targets and concepts for future drug development. Islet cell clusters were sensitive to the L-type Ca^2+^ channel blocker nifedipine. This maneuver resulted in a decrease of [Ca^2+^]_c_ as expected from numerous observations with murine β-cells and insulin-secreting tumor cell lines as well as the restricted number of studies with human β-cells. This shows that the biopsy material is suitable to receive reliable and reproducible results. This is also confirmed by the results observed with diazoxide used as gold standard in CHI treatment. Noteworthy, nifedipine has been used for the treatment of diazoxide-unresponsive CHI (19, 29), but due to reported hypotension in patients with mutations in the *ABCC8* gene, it is not commonly recommended for the treatment of CHI (29, 86).

Diazoxide is usually effective in all forms of CHI including severe cases caused by mutations in the genes encoding K_ATP_ channels (*ABCC8* and *KCNJ11*), e.g., in (2, 67, 87, 88). Our sample cohort was derived from patients harboring *ABCC8* missense mutations (patients 1, 6, and 7), a mutation affecting splicing (patient 3) and two nonsense mutations (patients 4,5). The missense and splicing mutations studied possibly allow production of SUR1 proteins albeit at reduced function or level. For nonsense mutation c.3970G>T p.(Glu1324*) detected in exon 32 of patient 4, nonsense-mediated RNA decay (NMD) has been predicted *in silico*, however, clinically the patient was reported to be diazoxide-responsive at dosage 8mg/kg/d. This may suggest escape of NMD with this particular mutation and generation of a truncated SUR1 protein lacking the last encoded six exons but retaining residual channel function. For one patient (patient 2) no mutation was found in the K_ATP_ channel genes (ABCC8 and KCNJ11) or in other CHI genes tested. This is not unusual in clinical routine. As this patient displayed a partial response to diazoxide (see **Table 1**) it was included in the study despite the unknown genetic background. Serious adverse drug effects are a consequence of the non-selectivity of diazoxide for pancreatic K_ATP_ (SUR1/Kir6.2) channels (89). Besides pancreatic K_ATP_ channels, the drug activates those of smooth muscles (SUR2B/Kir6.2 and SUR2B/Kir6.1) and exerts weak stimulatory effects on K_ATP_ channels of the cardiac muscle (SUR2A/Kir6.2) (90). One of the most common adverse effects of diazoxide, hirsutism, could be explained by activating both SUR1/Kir6.2 and SUR2B/Kir6.2 channels in hair follicles (91, 92).

#### NN414

As one strategy to improve CHI therapy we tested K_ATP_ channel openers more specific for β-cells. In comparison to diazoxide, NN414 is reported to be a selective SUR1 agonist, 100-fold more potent than diazoxide, suggesting that the drug is effective at much lower concentrations (62). Early, prediabetic stages of type 2 diabetes mellitus (T2DM) are normally characterized by compensatory hypersecretion of insulin. K_ATP_ channel openers have been suggested as beneficial medication to counteract excessive hormone release in prediabetic patients as insulin hypersecretion may cause or contribute to the development of glucose intolerance and β-cell degeneration in T2DM (93). NN414 has been used in numerous *in vitro* and *in vivo* studies to achieve β-cell rest, thereby preserving β-cell function and preventing apoptosis (94, 95). An animal *in vivo* study revealed a significant potential of NN414 in the treatment of disorders resulting from excessive insulin release (96). Alemzadeh et al. showed in a 6-week study that NN414 reduced hyperinsulinemia and improved glucose responsiveness in Zucker obese rats in a dose-dependent manner. NN414 entered human clinical trials for the treatment of T2DM. In healthy subjects, it inhibited insulin release, was well tolerated, and did not induce clinically relevant changes in safety parameters besides side effects on the gastrointestinal tract (97). NN414 was advanced in phase 2 of clinical trials where it showed a tendency to improve β-cell secretory function in diabetic patients (98, 99). The clinical trial was stopped because of elevated liver enzymes in treated patients (99, 100). The SUR1 selectivity, the low doses, and the reproducible Ca^2+^-lowering effect observed in our study in islet cell clusters from biopsy material (**Figures 2A, B**) suggest to consider NN414 as a potential alternative to diazoxide for the treatment of CHI with at least partially functioning K_ATP_ channels. Of note, this paper is not intended to characterize different types of CHI with respect to their diazoxide responsiveness or to recommend general treatment of all CHI types with NN414. Liver enzymes have to be monitored during therapy with NN414, but moderate elevation of their plasma concentration is no criterion to exclude the drug, although it would be desirable to develop NN414 analogues without this side effect. Notably, increased concentrations of circulating liver enzymes is one of the most reported side effects for octreotide that is used off-label as second-line therapeutic in the long-term management of CHI and for sirolimus that is proposed for patients resistant to diazoxide and octreotide (28, 101, 102), reviewed in (3).

#### VU0071063

Recently, a novel K_ATP_ channel activator, VU0071063 was discovered (70). VU0071063 is reported to be more selective for SUR1/Kir6.2 channels than for SUR2A/Kir6.2 and SUR2A/Kir6.1 channels. It has been demonstrated that it opens SUR1/Kir6.2 channels with a higher potency than diazoxide (70). VU0071063 was shown to activate K_ATP_ channels expressed in HEK-293 cells and to reduce glucose-stimulated Ca^2+^ influx in murine β-cells (70). Our data demonstrate at a first glance a beneficial characteristic of VU0071063 in human islet cell clusters isolated from CHI patients (**Figures 4A, B**), supporting the idea of a direct activation of K_ATP_ channels in pancreatic islets. By contrast, the observation that removal of VU0071063 from the solution initiated a transient drop in [Ca^2+^]_c_ suggests that the drug does not selectively interfere with K_ATP_ channels, but also with ATP production (71). This assumption is supported by the following observations: 1) VU0071063 strongly and reversibly depolarized ΔΨ in both, WT and SUR1^-/-^ islet cell clusters, which points to inhibition of ATP formation. 2) The removal of VU0071063 was followed by a transient drop in [Ca^2+^]_c_ in WT and SUR1^-/-^ islet cell clusters, which is presumably due to ATP-dependent SERCA activation. VU0071063 rapidly and significantly decreased [Ca^2+^]_c_ in SUR1^-/-^ islet cell clusters, too. This seems paradoxical as the ATP depletion leads to Ca^2+^ release out of the Ca^2+^ stores; however, the decreased [Ca^2+^]_c_ during application of the drug might be secondary to Ca^2+^-dependent inactivation of L-type Ca^2+^ channels. Our data suggest that the Ca^2+^-lowering effect of VU0071063 is caused by a dual mechanism: 1) direct opening of K_ATP_ channels and indirect opening of K_ATP_ channels by ATP depletion; 2) interference with SERCA function and Ca^2+^ release, thereby mediating unpredictable interactions with other ion channels. In conclusion, since VU0071063 raises the expectation of detrimental effects on mitochondria, thereby impairing all ATP-dependent processes, this compound seems not to be suitable for use in humans without structural modifications avoiding this side effect. Noteworthy, NN414 did not affect ΔΨ (compare **Figures 2E, F** to **4E, F**) and did hardly change [Ca^2+^]_c_ in SUR1^-/-^ islet cell clusters (compare **Figures 2C, D**to **4C, D**). These differences clearly show that the effects of NN414 are, in contrast to those of VU0071063, caused by a specific interference with K_ATP_ channels.

In summary, our data with K_ATP_ channel agonists demonstrate that these drugs can be effective in different forms of CHI caused by mutations in K_ATP_ channels. As shown by patch-clamp experiments (**Figure 3**), diazoxide and NN414 act as direct channel openers in mutated K_ATP_ channels with a dominant mutation comparable to WT channels. With respect to specificity, dosage, and expected side effects, NN414 seems superior to diazoxide. VU0071063 is unsuitable because of its multiple and yet not completely understood mode of action and therefore potential adverse side effects.

### Possible K_ATP_ Channel-Independent Strategies to Treat CHI

#### Current Second- And Third-Line Therapy Regimen Targeting K_ATP_ Channel Independent Pathways

There are mutations in *ABCC8* or *KCNJ11* genes known to disrupt the expression of K_ATP_ channels at the cell surface (4, 103, 104). In this case openers, e.g., diazoxide, are ineffective in the treatment of CHI (51, 105). For these patients it is indispensable to find drugs targeting mechanisms, which can induce β-cell rest and inhibit insulin release independent of K_ATP_ channels. Currently available alternatives to diazoxide therapy are somatostatin analogues (octreotide, octreotide-LAR, and lanreotide), sirolimus and exendin-(9–39) (3). Octreotide, a short-acting synthetic somatostatin analogue, inhibits insulin secretion by binding to and activating somatostatin receptors 2 and 5 (SSTR2 and SSTR5) (106). Activation of SSTRs shows multifactorial modulation of β-cells, which involves inhibition of the adenylate cyclase/cAMP pathway, activation of G protein-activated inwardly rectifying K^+^ (GIRK) channels, decrease in Ca^2+^ influx *via* P/Q-type Ca^2+^ channels and inhibition of exocytosis (24, 107, 108). Infants respond well to initial doses of octreotide, but tachyphylaxis after a few doses makes it not suitable for the long-term treatment. Long-acting somatostatin analogues (octreotide-LAR and lanreotide) have similar effects as octreotide but have the advantage that they are given once every 4 weeks, which improves therapy compliance and quality of life (21, 109, 110). However, due to a similar mechanism of action as octreotide, long-acting somatostatin analogues show similar side effects (111). Sirolimus, a mammalian target of rapamycin (mTOR) inhibitor, is an immunosuppressive and anti-proliferative agent that has been used in patients with diffuse CHI, unresponsive to diazoxide and octreotide therapy (22, 112). It suppresses insulin release by different mechanisms, which have not been fully elucidated (30). It has been proposed that downregulation of mTOR pathway decreases insulin production in pancreatic β-cells and β-cell growth and may restore ketogenesis (112, 113). Furthermore, upregulation of liver gluconeogenesis by sirolimus contributes to insulin resistance (114). However, severe and life-threatening side effects reported for the above-mentioned drugs restrict their use.

#### Opening of K_Ca_3.1 Channels

Beside K_ATP_ channels, Ca^2+^-activated K^+^ channels of intermediate conductance (K_Ca_3.1, SK4) contribute to K_slow_, the hyperpolarizing current that terminates bursts of action potentials in β-cells (74, 78). Accordingly, K_Ca_3.1 channels may become predominant regulators of membrane potential and insulin secretion when functional K_ATP_ channels are absent, which makes these channels ideal as drug targets in CHI. Our data show that an opener of K_Ca_3.1 channels, DCEBIO, was able to strongly reduce [Ca^2+^]_c_ in SUR1^-/-^ islet cell clusters. Furthermore, DCEBIO was highly effective in silencing human islet cell clusters obtained from pancreatic tissue of CHI patients (**Figure 5**). These experiments provide valuable support for the idea of targeting K_Ca_3.1 channels in the treatment of CHI. To follow this strategy would of course require the search for new K_Ca_3.1 channel openers with high selectivity for β-cells since unspecific K_Ca_3.1 channel openers are expected to exert severe side effects in numerous organs (e.g., lung, cells of the hematopoietic system, and salivary glands) (115–117).

#### DXM as L-type Ca^2+^ Channel Antagonist

The block of NMDA receptors by DXM is expected to increase insulin secretion (81). However, DXM has a higher affinity for L-type Ca^2+^ channels than to its known target, the NMDA receptor (118). As mentioned before, DXM moderately inhibits L-type Ca^2+^ channels, thereby lowering [Ca^2+^]_c_ in permanently depolarized β-cells under stimulating glucose concentrations (82, 119). This is exactly what we observe in our experiments with depolarized islet cell clusters isolated from pancreatic tissue of CHI patients and with depolarized islet cell clusters from SUR1^-/-^ mice (**Figure 6**). Thus, DXM could be an alternative strategy for the treatment of CHI especially in the diffuse form of CHI. In patients with focal lesions, where only a distinct portion of β-cells, i.e., the focal ones, is dysregulated, the drug could cause an undesired increase in [Ca^2+^]_c_ and insulin secretion in healthy islets. The benefits of this drug are that it is already available as a pharmaceutical and that inhibition of L-type Ca^2+^ channels by e.g., nifedipine is already a proved treatment of CHI (19, 29). Considering side effects of nifedipine, like hypotension, the moderate effect of DXM on L-type Ca^2+^ channels could be of advantage (82).

#### Simvastatin

It was shown that simvastatin lowers insulin secretion by blocking L-type Ca^2+^ channels (84, 85). The effect of simvastatin on [Ca^2+^]_c_ was comparable with that of nifedipine in the insulin-secreting cell line MIN-6 (85). This interaction might contribute to the increased risk to develop diabetes mellitus under a cholesterol-lowering therapy with statins (83). With respect to CHI patients, this side effect could constitute a suitable therapeutic approach. Our results obtained from experiments with islet cell clusters isolated from SUR1^-/-^ mice and human islet cell clusters isolated from patients with CHI indeed point toward a possible beneficial effect of simvastatin in the treatment of CHI (**Figure 7**). Noteworthy, statins are widely used and well tolerated in the long-term therapy. In contrast to nifedipine, which affects the cardiovascular system, statins are safe with respect to blood pressure or heart rate (120). Additionally, it is proposed that statins induce hepatic gluconeogenesis in human liver cells by activation of the pregnane X receptor (PXR) (121, 122), which could also counteract hypoglycemic conditions in CHI patients.

## Conclusions

There is a clear need to develop novel approaches to prevent hypoglycemia in CHI patients and to establish better therapies with less side effects for the different forms of CHI. In this study, we had access to biopsy material of CHI patients and give suggestions, which drugs or targets should be studied in future. Promising results were obtained for NN414, DCEBIO, DXM and simvastatin.

## Data Availability Statement

The raw data supporting the conclusions of this article will be made available by the authors upon request.

## Ethics Statement

Ethics approval for the study involving human participants was approved by the ethic commission of the Universitätsmedizin Greifswald (BB 050/13). Written informed consent was provided by the legal guardians of the children for the study. The animal study was reviewed and approved by the Ethics Committee of Regierungspraesidium Tuebingen.

## Author Contributions

JS researched the data and wrote and edited the manuscript. TH, AG, and JK researched the data, contributed to the discussion, and edited the manuscript. WB, CW, and UL provided human β-cells and contributed to discussion. IW supported the manuscript as an expert in CHI genetics and contributed to the discussion. MD and PK-D contributed to discussion and study design and edited the manuscript. GD designed the study, wrote and edited the manuscript, and contributed to discussion. GD is the guarantor of this work and, as such, had full access to all the data in the study and takes responsibility for the integrity of the data and the accuracy of the data analysis. All authors contributed to the article and approved the submitted version.

## Funding

This work was supported by grants from the DFG (DR225/11-1 to GD and INST 211/647-1 FUGG to MD).

## Conflict of Interest

The authors declare that the research was conducted in the absence of any commercial or financial relationships that could be construed as a potential conflict of interest.
